# Deep learning time series prediction models in surveillance data of hepatitis incidence in China

**DOI:** 10.1371/journal.pone.0265660

**Published:** 2022-04-13

**Authors:** Zhaohui Xia, Lei Qin, Zhen Ning, Xingyu Zhang

**Affiliations:** 1 National Enterprise Information Software Engineering Research Center, School of Mechanical Science and Engineering, Huazhong University of Science and Technology, Wuhan, China; 2 School of Computer Science and Technology, Huazhong University of Science and Technology, Wuhan, China; 3 Starzl Transplant Institute, University of Pittsburgh Medical Center, Pittsburgh, PA, United States of America; Newcastle University, UNITED KINGDOM

## Abstract

**Background:**

Precise incidence prediction of Hepatitis infectious disease is critical for early prevention and better government strategic planning. In this paper, we presented different prediction models using deep learning methods based on the monthly incidence of Hepatitis through a national public health surveillance system in China mainland.

**Methods:**

We assessed and compared the performance of three deep learning methods, namely, Long Short-Term Memory (LSTM) prediction model, Recurrent Neural Network (RNN) prediction model, and Back Propagation Neural Network (BPNN) prediction model. The data collected from 2005 to 2018 were used for the training and prediction model, while the data are split via 5-Fold cross-validation. The performance was evaluated based on three metrics: mean square error (MSE), mean absolute error (MAE), and mean absolute percentage error (MAPE).

**Results:**

Among the year 2005–2018, 20,924,951 cases and 11,892 deaths were supervised in the system. Hepatitis B (HB) is the most disease-causing incidence and death, and the proportion is greater than 70 percent, while the percentage of the incidence and deaths is decreased much in 2018 compared with 2005. Based on the measured errors and the visualization of the three neural networks, there is no one model predicting the incidence cases that can be completely superior to other models. When predicting the number of incidence cases for HB, the performance ranking of the three models from high to low is LSTM, BPNN, RNN, while it is LSTM, RNN, BPNN for Hepatitis C (HC). while the MAE, MSE and MAPE of the LSTM model for HB, HC are 3.84*10^−06^, 3.08*10^−11^, 4.981, 8.84*10^−06^, 1.98*10^−12^,5.8519, respectively.

**Conclusions:**

The deep learning time series predictive models show their significance to forecast the Hepatitis incidence and have the potential to assist the decision-makers in making efficient decisions for the early detection of the disease incidents, which would significantly promote Hepatitis disease control and management.

## Introduction

Hepatitis is an inflammation of the liver tissue, and it is a worldwide disease with a high mortality rate [1], which can sometimes progress to fibrosis, cirrhosis or liver cancer. The most common causes of hepatitis worldwide are viruses, while other causes involve heavy alcohol use, toxins, autoimmune diseases, etc. [2]. There are five main types of viral hepatitis: type A, B, C, D and E, while all of them cause liver disease in different ways [2]. Hepatitis A (HA) is an infectious disease of the liver caused by Hepatovirus A, presents in the feces of infected people, and is often transmitted via consumption of contaminated food or water (via fecal-oral route). The incubation period of HA is around 2–6 weeks [3]. Hepatitis B (HB) is an infectious disease caused by the hepatitis B virus affecting the liver, which is transmitted via exposure to infective blood, semen, and other body fluids, while it can be transmitted from infected mother to baby during pregnancy or childbirth. The incubation period of HB is about 30 to 180 days [4]. Hepatitis C (HC) is an infectious disease caused by the hepatitis C virus mainly influencing the liver, which is mostly transmitted via infective blood, while less possibility via sexual transmission. The incubation period of HC is around 1 to 3 months [5]. Hepatitis D (HD) can only infect people already infected with hepatitis B [6], while Hepatitis E (HE) is inflammation of the liver caused by the infection from hepatitis E virus, which is mainly transmitted via consumption of contaminated water or food (via fecal-oral route). The incubation period of HE is about 15 to 60 days [7]. HA and HE behave similarly that do not lead to chronic hepatitis, which are common in developing countries. HB and HC can be either acute or chronical, while HB infection is most commonly self-limiting in adults and frequently leads to chronic infection in kids, but HC is usually leads to chronic infection [8]. According to the World Health Organization (WHO), viral hepatitis caused 1.34 million deaths, and the number of deaths due to hepatitis is increasing, while the majority of the deaths are caused by chronic liver disease (0.72 million by cirrhosis and 0.47 by hepatocellular carcinoma) in 2015 [9]. Hepatitis leads to more than a million deaths a year while most of the deaths are indirectly caused by liver scarring or liver cancer [10]. In some underdeveloped areas, Hepatitis is still a life-threatening infectious disease, while the occurrence of infectious diseases having their own rules is often influenced by the speed of pathogen variation, accumulation of susceptibility, and environmental changes [11]. Early identification of epidemic rules is vital for the prevention and hepatitis control [12].

Therefore, public health surveillance systems are established to facilitate the monitoring of infectious diseases, while the goal is to monitor and forecast the trends to minimize morbidity and mortality [13]. Different statistical methods are proposed for predicting infectious disease incidence [14–17]. Among these models, there are some drawbacks for time series analysis and regression analysis to find out the epidemic rules due to their relationship complexity [12, 18]. Artificial neural networks (ANN) can approximately identify the rules due to the characteristics of robustness, fault tolerance, and adaptive learning ability, thus they have been widely adopted for time series forecasting to efficiently obtain nonlinear relationships from the data [19, 20]. Among ANN models, there are three models are commonly adopted methods for classification and nonlinear regression problems: the back-propagation neural network (BPNN), the recurrent neural network (RNN), and the long short-term memory (LSTM) [21–23]. BPNN is a type of backward propagation of errors and multilayered feed-forward neural networks, which is commonly adopted in engineering, weather prediction areas, etc.[24, 25]. There are also some scholars adopting BPNN to predict Hepatitis A incidence [12].

RNN is a sub-class of ANN using hidden variables as a memory to capture temporal dependencies between system and control variables, which is widely adopted in sequence learning problems [26] and language processing with good performance [27]. LSTM is a type of RNN comprising a cluster of recurrent connected subnets to allow it can deal with the exploding and vanishing gradient problems, which is widely used in handwriting recognition, and voice recognition, etc. [28, 29]. Autoregressive integrated moving average(ARIMA), support vector machine(SVM) and LSTM recurrent neural network were adopted to predict Hepatitis E and compared [30]. A new method for the Hand-foot-mouth disease (HFMD) prediction using GeoDetector and a LSTM is proposed to predict the incidence of HFMD [31]. A forecasting model of the COVID-19 outbreak in Canada using state-of-the-art Deep Learning (DL) models is developed to predict the trends and possible stopping time of the current COVID-19 outbreak around the world [32]. A new artificial intelligence (AI) model, viz., Sentiment Informed Time-series Analyzing AI (SITALA), trained on COVID-19 test positivity data and news sentiment from over 2750 news articles for Harris county is introduced [33].

However, there is rare research focusing especially on the applicability of predicting infectious diseases e.g., Hepatitis with RNN and LSTM. Therefore, motivated by the advantages of the LSTM model, this paper aims to predict the Hepatitis incidence in mainland China. To obtain the goal, BPNN, RNN and LSTM models have been used to predict Hepatitis disease incidence, and the forecasting abilities of the models were compared to seek the best-matching time series modeling technique for Hepatitis, which will be possible for the government to forecast the trend of Hepatitis incidence and deaths and prepare effective intervention measures for Hepatitis prevention at an early stage.

In this paper, we described the epidemiological trend of hepatitis disease from 2005 years to 2018 years in China for the first time. We also introduced and compared three typical deep learning methods in the prediction of hepatitis incidence based on the infection surveillance data.

## Materials and methods

### Materials: CDC data

The Hepatitis monthly incidence data are gathered from the Chinese Center for Disease Prevention and Control (CDC). The internet-based surveillance system of China was established in 2004 covering the largest surveillance population in the world. The Chinese Government strengthened its overall public health disease surveillance following the establishment of the national surveillance system, while the surveillance system covers 39 notifiable infectious diseases reported to the network [34]. The incidence time series of Hepatitis A (HA), Hepatitis B (HB), Hepatitis C (HC), Hepatitis E (HE), and Hepatitis U (other Hepatitis) in the whole country are collected by CDC and published every month. The data we collected are from 2005 to 2018.

### Neural networks based models

**Fig 1:**
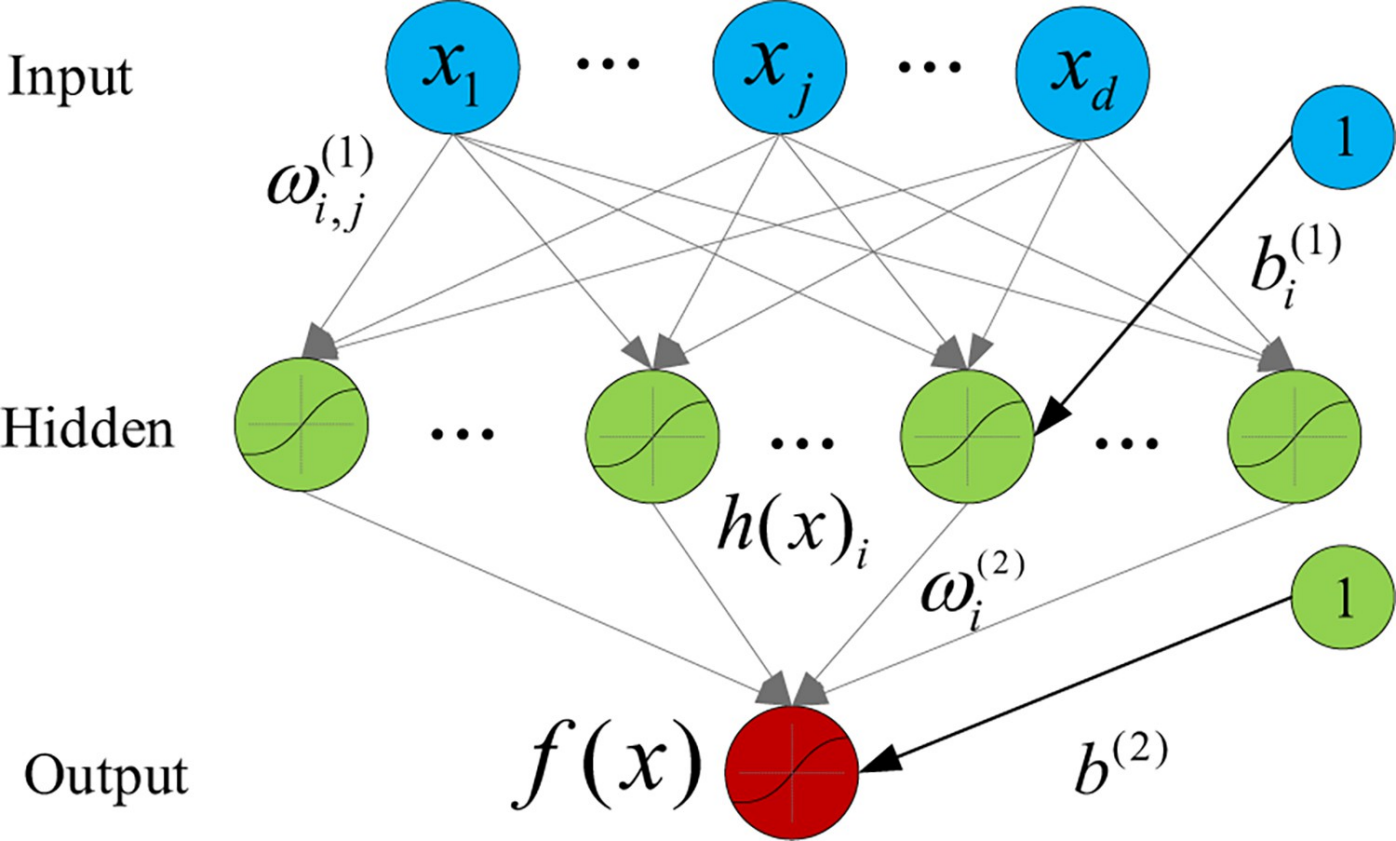
BPNN model.

Artificial neural networks (ANN) were created to imitate the features of the biological neurons in the human brain and nervous system, and they keep the biological concept of artificial neurons [35]. ANN consists of initial input data, activation function, and producing output with an output function, while the activation function can provide a smooth transition as input values modify [36]. The ANN is composed of connections, while each connection is indicated a weight as its related importance, which can provide the output of one neuron as the input of another neuron [37]. In the Hepatitis forecasting modeling, the historical incidence is used as the input neurons, while the related predicting incidence is obtained from the output neurons once the ANN is properly trained. The ANN can learn the information involved in the historical incidence series via modifying the connection weights. There are several advantages that ANN has for predicting time series data, e.g., having the capabilities to fully extract the complex nonlinear relationships hidden in the time series data. Here are the theories of three types of ANN:

Back-propagation neural networks (BPNN). BPNN is a type of feed-forward ANN, in which the information transmits in only one direction from the input neurons through the hidden neurons and to the output neurons. A single hidden layer BPNN includes an input layer, a hidden layer and an output layer as shown in Fig 1.

The BPNN, which is a branch of ANN, is a type of feedforward neural network [38]. In this network, the data moves in only one direction from the input neurons to the output neurons through the hidden neurons, which have no cycles or loops. There are three layers in BPNN: input layer, hidden layer and output layer. Each layer consists of some neurons. The neurons in the forward layers are fed directly to the back layer via a series of weights *ω*_*i*,*j*_ and *ω*_*j*_, in which *i* = 1,2,…,*n*, *j* = 1,2,…*m*, while n represents the neuron number of the input layer and m indicates the neuron number of the hidden layer. The architecture of a BPNN model can be demonstrated as in Fig 1. In this paper, the Hepatitis incidence data are trained in the BPNN model with a back-propagation algorithm, the training samples are stored in the input layer. The outputs can be obtained via the related functions and connection weights between the neurons in the different layers. During the training process in the BPNN model, the error should meet the pre-set accuracy requirements. The weights between the neurons will be adjusted automatically along the reverse direction of the BPNN until the minimum network error up to the criterion [39].

For training the BPNN model, there are three major steps which involve forwardly feeding the networks with the input data, computing the network error and back-propagating the error, adjusting the connection weights between the neurons.


$$\begin{array}{l} B_{j}=\overset{n}{\underset{i=0}{\sum }}\omega_{ij}x_{i}+\theta_{j}(i=0,1,\ldots ,n;j=1,2,\ldots ,m)\\ y_{j}=f(B_{j})(j=1,2,\ldots ,m) \end{array}$$ (1)

In which, *ω*_*i*,*j*_ is the connection weights from neuron *i* in the input layer to neuron *j* the hidden layer, *B*_*j*_ represents the activation value of the *j*th neuron in the hidden layer, *θj* is an additional bias term, *x*_*i*_ is the *i*th input while *y*_j_ indicates the output of the *j*th neuron, *f* represents the activation function of a neuron, which is often a sigmoid function.


$$f(t)=\frac{1}{1+e^{-x}}$$ (2)

The output T of all output layer neurons can be described as:

$$T=f(\sum_{j=0}^{m}\omega_{j},y_{j})(j=0,1,2,\ldots m)$$ (3)

In which *w*_*j*_ is the connection weight from the neuron j in the hidden layer to the output neuron, while *y*_*j*_ is the output value of the neuron j in the hidden layer. The weights between the neurons are random in the initial state, and they will be adjusted based on the BPNN training results. Many approaches can be used for the weights adjustment, e.g., Newton’s method, Gauss-Newton’s algorithm, steepest descent algorithm and Levenberg-Marquardt algorithm, etc.[40]. In this paper, Levenberg-Marquardt algorithm is adopted since it inherits the advantages with speed and stability from other methods. The frame TensorFlow with Python is used for implementing BPNN due to its plentiful effective toolbox for neuron networks. The BPNN model and its corresponding training algorithms can be easily developed using the TensorFlow frame [41].

### RNN models

**Fig 2:**
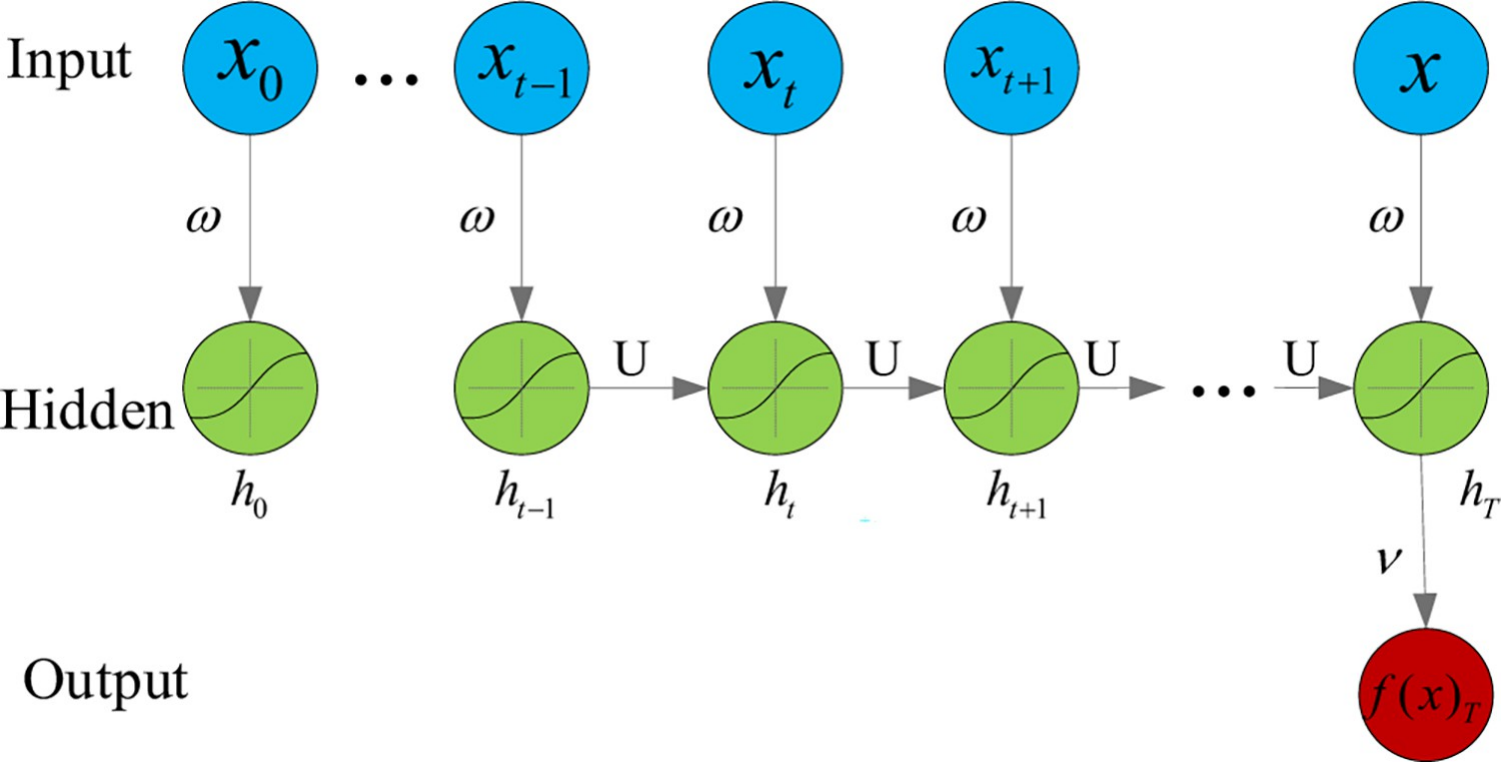
Basic recurrent neural network (RNN) model.

The RNN is a type of ANN, which has better capabilities to capture temporal dependencies especially benefits making t-step ahead predictions. The t time step forecasting depends on the present state and all control actions in a time series *t*∈{0,…,*s*−1}, similarly, predicting the time step t-1 depends on the present state and all previous actions in time step *t*∈{0,…,*s*−2}. The structure of a basic RNN is demonstrated with compact and unfolded forms as Fig 2. Each layer consists of a few cells, while each cell represents a time-step. The state of the previous time-step *s*∈{0,…,*t*−1} serves as the input for the time-step s+1. In each cell, there is N number of hidden neurons encoding the state representation [42, 43]. A single RNN cell in a one-layer RNN can be expressed mathematically as:

$$\begin{array}{l} h_{t-1}=\omega_{x,h}x_{t-1}+\omega_{h,h}h_{t-1}+b_{1}\\ y_{t}=f(h_{t}+b_{2}) \end{array}$$ (4)

In which, *t*∈{1,…,*s*} is the time series index while *s* is the prediction horizon, *h*_*t*_ indicates the state of the cell in the hidden layer for the time-step *t*. *x*_*t*_-1 represents the inputs while *y*_*t*_ the outputs corresponding to the prediction time-step *t*. *b*_1_ and *b*_2_ are the bias, *ω*_*x*,*h*_ and *ω*_*h*,*h*_ are the weight from the neuron in the input layer to the neuron in the hidden layer and the neurons in the hidden layer respectively. *f* is the activation function, *h*_0_ is the initial state.

A deep RNN with l-layers greater than one layer still can be illustrated by Eq (4), while deep RNN has the more enhanced capabilities to learn the features from the dynamical hierarchical system. But RNN often takes a longer training time which prevents the data from having more parameters to train. An RNN is characterized by the values of *ω*_*x*,*h*_, *ω*_*h*,*h*_, *b*_1_ and *b*_2_, etc. for each layer. These parameters are adjusted based on the training results via minimizing the forecasting error of the RNN model through a user-defined loss function. During the training process, a back-propagation through time algorithm is adopted to evaluate the gradient of the loss function as an optimizing algorithm to modify the connection weights among the neurons in different layers. The adaptive moment estimation algorithm (Adam) [44] is widely used as the optimizing algorithm for RNN training.

### LSTM models

**Fig 3:**
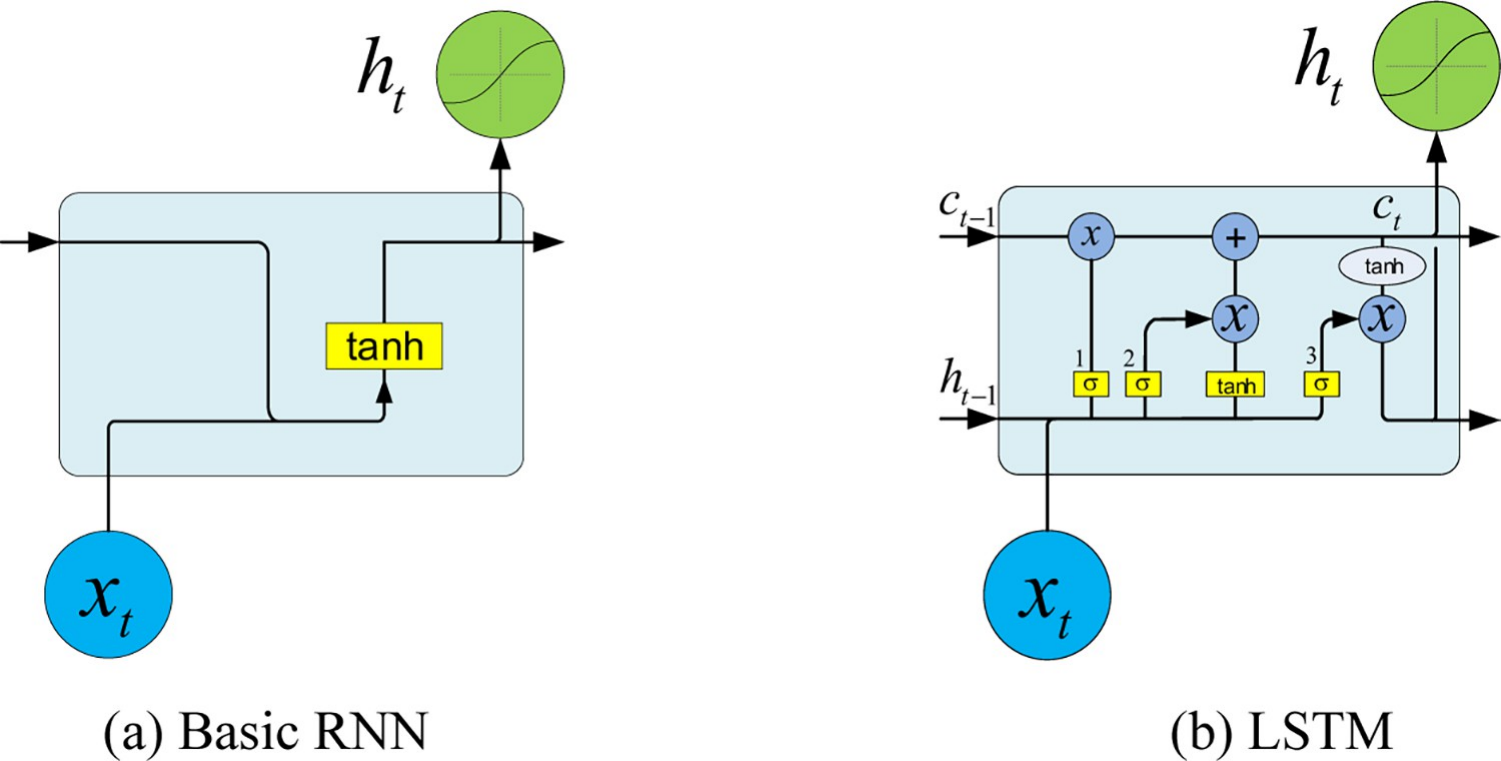
RNN models. (a) Basic RNN, (b) LSTM.

LSTM is an RNN architecture used in the field of deep learning, while the cell of RNN is shown in Fig 3(a). Compared with the standard feedforward neural networks, LSTM has feedback connections, which makes it has the capabilities to process single data points like images and entire sequence data like speech or videos. The powerful characters make LSTM can predict diseases. A common LSTM consists of a cell, an input gate, an output gate and a forget gate as shown in Fig 3(b), while the cell can remember values over arbitrary time intervals and the data flow can be regulated into and out of the cell [31, 45].

Intuitively, the cell keeps track of the dependencies among the elements of the input sequence. The input gate takes charge which new value flows into the cell. The forget gate is responsible to determine which value remains in the cell while the output gate controls which value should be sent to output activation, which is usually a logistic sigmoid function. In LSTM models, the input gates are connected with the out gates, while a few are recurrent. The connection weights will be adjusted during the training process which determines how the three gates operate. The compact forms of LSTM can be illustrated mathematically as:

$$\begin{array}{l} f_{t}=\sigma_{g}(\omega_{f}x_{t}+u_{f}h_{t-1}+b_{f})\\ i_{t}=\sigma_{g}(\omega_{i}x_{t}+u_{i}h_{t-1}+b_{i})\\ o_{t}=\sigma_{g}(\omega_{o}x_{t}+u_{o}h_{t-1}+b_{o}) \end{array}$$ (5)

In which the bolder variables represent vectors, ***b*** is bias vector, matrices *ω* and u are the input and recurrent connection weights respectively, which will be adjusted from the training process. The subscript *i*, *o*, ***f*** and ***c*** indicate input gate, output gate, forget gate and the memory cell separately. *t* indicates the index of the time-step. *x*_*t*_ is the input vector, ***f***_*t*_ is the activation vector of the forget gate, it is the activation vector of the input/update gate, *o*_*t*_ represents the activation vector of the output gate. ***h***_*t*_ is the state vector in the hidden layer which is also the output vector of the LSTM unit. ***c***_*t*_ is the cell state vector. *σ*_*g*_ is an activation function with a sigmoid function.


$$\begin{array}{l} c_{t}=f_{t}\circ c_{t-1}+i_{t}\circ \sigma_{c}(\omega_{c}x_{t}+u_{c}h_{t-1}+b_{c})\\ h_{t}=o_{t}\circ \sigma_{h}(c_{t}) \end{array}$$ (6)

In which the operator o represents the Hadamard product [46], the initial values are h_0_ = 0 and ***c***_0_ = 0. *σ*_*c*_ is an activation function with hyperbolic tangent function, while *σ*_*h*_ is an activation function with hyperbolic tangent function. A neural network with LSTM units can be trained with training sequence data using an optimizing algorithm such as gradient descent combing with BPTT to calculate the gradients.

### Cross-validation

Cross-validation is a measurement to test the ability of a predictive model to predict new data that was not used in estimating it, while the results of a statistical analysis will generalize to an independent dataset [47]. There are many types of cross-validation, e.g., leave-*p*-out cross-validation validation, leave-one-out cross-validation, repeated random sub-sampling validation, holdout method, *K*-fold cross-validation, and so on. The *K*-fold cross-validation, a technique of randomly dividing the original sample into *K* equal-sized subsamples, for choosing parameters of the model is adopted herein [48]. For the *K* subsamples, a single subsample is retained as the validation data to test the model, while the remaining *K*-1 subsamples for training the model. The cross-validation process is then repeated K times, and the *K* results can then be averaged to produce a single estimation. A typical process of estimating a turning parameter *λ* with K-fold cross-validation is as follows: (1) approximately dividing the sample dataset *D*_*n*_ into *K* equal subsamples *S* = {*S*_1_, *S*_2_,…,*S*_*K*_}; (2) For each subsample *S*_*i*_, it will fit the model with a parameter to other K-1 subsamples, giving ${\hat{\tau }}^{‐k}(\lambda )$ and compute its error in predicting the *k*th subsample as in Eq (7):

$$E_{k}(\lambda )=\sum_{i=1}^{K}{\left(y_{i}-x_{i}{\hat{\tau }}^{-k}(\lambda )\right)}^{2}$$ (7)

The cross-validation error can be expressed as:

$$\sigma (\lambda )=\frac{1}{K}\sum_{i=1}^{K}E(\lambda )$$ (8)

Additionally, the cross-validation should be used very carefully due to its data leakage and overfitting[49, 50]. In this paper, *K* = 5 is adopted to train the model, while *λ* is chosen to make *σ*(*λ*) smallest.

### Model selection criterion and evaluation index

In ANN, the modeling data is usually split into two groups: training data for training the data, while validation data for testing the model efficiency based on ANN. The selection of the best model based on ANN is determined via the minimization of the bias between the values gained from the training and validation data and the values in the raw data. The comparison between the forecasted value of the three approaches based on ANN and the observed value from the raw data is adopted to determine the efficiency of the three predicting approaches in this research. The mean absolute error (MAE), mean absolute percentage error (MAPE), and the mean square error (MSE) are adopted as the evaluating measures, which are commonly adopted in selecting predictions to measure the accuracy and bias of models [16, 51], which can be expressed mathematically as:

$$MAE=\frac{1}{n}\sum_{t=1}^{n}\left|y_{t}-x_{t}\right|$$ (9)

$$MAPE=\frac{100\%}{n}\sum_{t=1}^{n}\left|\frac{y_{t}-x_{t}}{x_{t}}\right|$$ (10)

$$MSE=\frac{1}{n}\sum_{t=1}^{n}{\left(y_{t}-x_{t}\right)}^{2}$$ (11)

Where *y*_*t*_ is the forecasted values at time-step *t*, *x*_*t*_ is the observed value of the raw data at time-step *t*, while *n* is the number of forecasting.

## Results

### Development and results of neural networks

Three artificial neural networks were adopted herein to fit the incidence and death trend of Hepatitis. The available incidence/death time series were divided into different subsamples as K-fold cross-validation need. The optimum neural networks were obtained based on the least MSE between the training and test datasets.

The number of cases of the 5 class hepatitis diseases from 2005–2018 is listed in Table 1. Hepatitis B is the highest incidence of hepatitis. The number of deaths from 2005 to 2018 caused by the different types of hepatitis is listed in Table 2.

**Table 1:** Numbers of cases caused by hepatitis diseases in China.

	HA	HB	HC	HE	HU	H
2005	76102	1132805	59159	15397	82969	1366432
2006	70889	1261735	77315	18455	78907	1507301
2007	79349	1327225	100258	20513	75715	1603060
2008	58820	1330654	118201	19679	63824	1591178
2009	45372	1330352	141609	20854	55556	1593743
2010	36250	1193266	163174	24260	51331	1468281
2011	32659	1252236	188807	30459	50318	1554479
2012	25452	1257320	219110	29859	41979	1575588
2013	22891	1113319	223094	28991	39321	1427626
2014	26740	1084543	222528	27943	34804	1396558
2015	23418	1085113	232400	27986	29518	1398435
2016	21866	1100691	231725	28671	24699	1407652
2017	19603	1180545	242897	29844	21201	1494090
2018	16736	1225877	251246	29435	17234	1540528

**Table 2:** Numbers of deaths caused by hepatitis diseases in China.

	HA	HB	HC	HE	HU	H
2005	36	849	102	44	103	1134
2006	33	841	151	40	88	1153
2007	23	838	123	39	75	1098
2008	13	930	131	31	59	1164
2009	22	830	155	24	41	1072
2010	6	723	142	34	32	937
2011	14	686	137	41	18	896
2012	9	638	110	23	23	806
2013	4	593	163	20	19	789
2014	8	398	134	15	13	568
2015	12	353	98	12	6	481
2016	6	430	111	18	5	570
2017	4	455	129	27	3	618
2018	4	470	115	15	2	606

**Fig 4:**
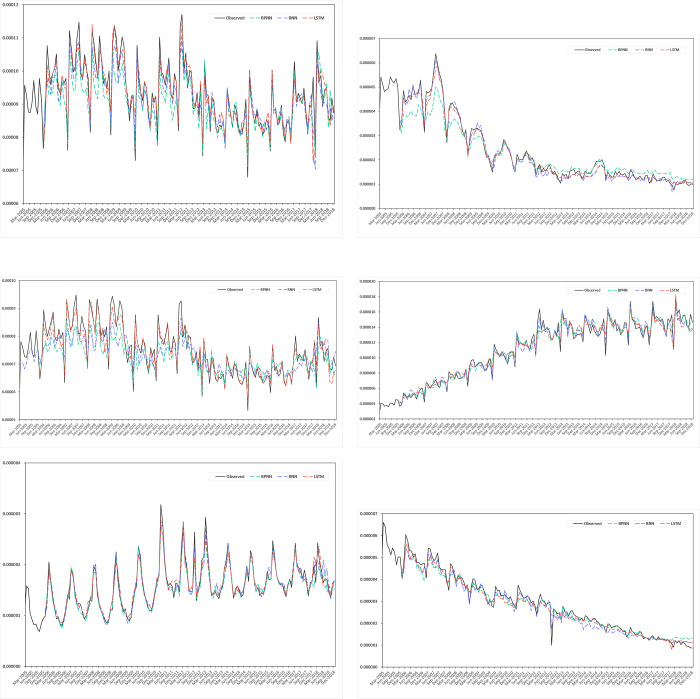
Incidence and fitting values of Hepatitis predicted by three neural network models. (a) Hepatitis, (b) Hepatitis A, (c) Hepatitis B, (d) Hepatitis C, (e) Hepatitis E, (f) Hepatitis U.

Considering the number of cases between 2015 to 2018, hepatitis B is the highest proportion as shown in Fig 4, while hepatitis C is the second-highest proportion of hepatitis diseases. In this research, three different ANNs are employed to fit the hepatitis incidence trend. To estimate how accurately a predictive model will perform in practice, cross-validation is adopted to split the training and test data, which is mainly used. The goal of cross-validation is to test the model’s ability to predict new data, flag overfitting or selection bias issues, and give how the model generalizes to an independent dataset. K-fold cross-validation can provide a solution that divides the dataset into different folds and makes each fold have some point as a testing dataset, which divides a given dataset into a K number of folds [52].

The number of inputs of the neural networks will not require any transformation of the original incidence series for the training. In this study, the number of neurons in the input layer of BPNN, RNN and LSTM neural networks are set to the same number as the *lookback* value as in Eq 12, which is a parameter that presents the number of values in each row (e.g., using the data from January to December in 2005 to predict the data of January in 2006, then *lookback* = 12). The output layer of the neural networks is only one neuron indicating the forecast value of the incidence of the next month. The input matrix and the corresponding output matrix of the training and test sample herein can be expressed as:

$$\Gamma =[\begin{array}{llllllllllll} x_{1} & x_{2} & x_{3} & x_{4} & x_{5} & x_{6} & x_{7} & x_{8} & x_{9} & x_{10} & x_{11} & x_{12}\\ x_{2} & x_{3} & x_{4} & x_{5} & x_{6} & x_{7} & x_{8} & x_{9} & x_{10} & x_{11} & x_{12} & x_{13}\\ \cdots & \cdots & \cdots \\ x_{t-12} & x_{t-11} & x_{t-10} & x_{t-9} & x_{t-8} & x_{t-7} & x_{t-6} & x_{t-5} & x_{t-4} & x_{t-3} & x_{t-2} & x_{t-1} \end{array}]$$ (12)

$$\Phi =[\begin{array}{l} x_{13}\\ x_{14}\\ \ldots \\ x_{t} \end{array}]$$ (13)

In which, Г and Φ represent the input matrix and corresponding output matrix respectively, while the number of elements in each row can be changed. In this paper, two different *lookback* values (the incubation period and 12) are adopted for HA, HB, HC and HE (while the average incubation period are 1, 3, 2 and 1 months respectively). *x*_*t*_ indicates the sample value at time *t*. Г is transferred into the input layer for training, while Φ is its training goal. These matrices are then placed into Python neural network functions, and the corresponding parameters are appropriately set.

In artificial neural networks, the computational efficiency and accuracy are influenced by not only learning rates and algorithms but also the number of neurons in the hidden layers. There are no standard rules for adopting the number of layers and neurons, while it can be optimized via multiple trials and model error [53].

The learning rates are tested from 0.0015 to 0.05 with 0.0005 increments for examination. Based on test, the learning rates have a little influence for the result, thus 0.0025 is adopted herein. The number of neurons in the hidden layer was tested from 3 to 12 with 3 increment for each method.

In BPNN,.. For RNN,. For LSTM,.

### Comparisons of forecasting performance

The incidence and death values caused by Hepatitis diseases including Hepatitis A, Hepatitis B, Hepatitis C, Hepatitis E, Hepatitis U (representing other Hepatitis diseases) in China from 2005 to 2018 are listed in Tables 1 and 2. Among different Hepatitis diseases, the number of cases caused by HB is the largest. The number of HA cases has been approximately decreased year by year, and it is lower than the number of HC, HE and HU in 2018. However, the number of HC cases is generally increasing every year, and it is the second Hepatitis disease-causing incidence and deaths.

The proportion of incidence and death cases for the different diseases from 2005 to 2018 are illustrated below. Hepatitis B is the most disease-causing incidence and death, and the proportion is greater than 70 percent. However, the percentage of the incidence and deaths is decreased much in 2018 compared with 2005. The percentage of incidence and deaths caused by HC is gradually growing year by year, and the percentage is up to 16.3% and 19% respectively. But after 2016, it keeps in a similar percentage. In 2005, the lowest number of incidence and death cases are Hepatitis E and Hepatitis A respectively, while in 2018, they are Hepatitis A and Hepatitis U.

Fig 4(a) shows the incidence and fitting values of Hepatitis predicted by three neural network models, as well as the observed values, while Fig 4(b) to Fig 4(f) indicate the incidence and fitting values of Hepatitis A, Hepatitis B, Hepatitis C, Hepatitis E, Hepatitis U, respectively by the different neural network models. The figures show that the predicted values in the three models matched the observed data measurably.

**Fig 5:**
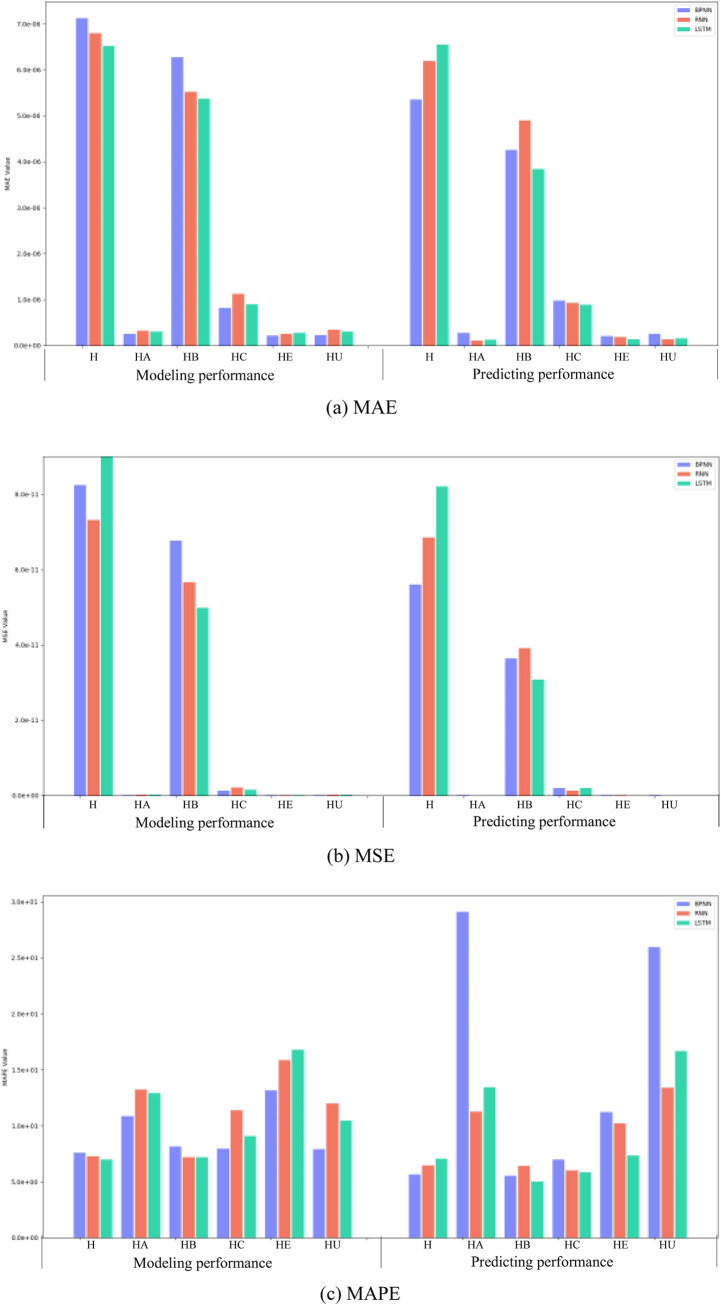
Prediction performance of the three neural network models. (a) MAE, (b) MSE, (c) MAPE.

Table 3 and Fig 5 show the modeling and prediction performance of the three neural network models. From the figure, the MAE, MAPE and MSE measures in the LSTM model are the lowest in the training performance, but not in the predicting performance. Based on the measured errors and the visualization of the three neural networks, there is no one model predicting the incidence cases that can be completely superior to other models. When predicting the number of incidence cases for HB and HC (they are the most common two Hepatitis diseases in recent years), the performance ranking of the three models from high to low is LSTM, BPNN, RNN. For HE predicting, the ranking is LSTM, RNN, BPNN, while it is BPNN, RNN, LSTM for HU predicting. However, the ranking is BPNN, LSTM, RNN to forecast the whole Hepatitis disease incidence cases. We have the lookback value (about to the incubation period, and 12) for HA, HB, HC and HE, while only *lookback* = 12 only for H and HU (since there are no incubation period for them).

**Table 3:** Comparison among different models.

Type	Models	*lookback*	Simulated performance			Predicted performance		
			MAE	MSE	MAPE	MAE	MSE	MAPE
H	BPNN	12	7.12*10^−06^	8.25*10^−11^	7.5918	5.35*10^−06^	5.60*10^−11^	5.6589
	RNN	12	6.79*10^−06^	7.32*10^−11^	7.2593	6.19*10^−06^	6.86*10^−11^	6.4674
	LSTM	12	6.52*10^−06^	9.01*10^−11^	6.9687	6.55*10^−06^	8.21*10^−11^	7.0667
HA	BPNN	1	6.80*10^−07^	1.00*10–12	29.6218	1.32*10–06	1.76*10^−12^	134.4940
	BPNN	12	2.58*10^−07^	1.57*10^−13^	10.8548	2.79*10^−07^	8.68*10^−14^	29.1055
	RNN	1	4.30*10^−07^	4.50*10^−13^	17.4566	4.51*10^−07^	6.18*10^−13^	50.7638
	RNN	12	3.25*10^−07^	2.21*10^−13^	13.2379	1.04*10^−07^	1.79*10^−14^	11.2512
	LSTM	1	4.50*10^−07^	5.30*10^−13^	17.5645	1.08*10^−07^	2.45*10^−14^	11.6492
	LSTM	12	3.06*10^−07^	1.83*10^−13^	12.9311	1.24*10^−07^	2.31*10^−14^	13.4330
HB	BPNN	3	5.95*10^−06^	5.81*10^−11^	7.8670	5.46*10^−06^	5.41*10^−11^	7.2589
	BPNN	12	6.27*10^−06^	6.77*10^−11^	8.1580	4.26*10^−06^	3.64*10^−11^	5.5301
	RNN	3	6.55*10^−06^	6.94*10^−11^	8.7680	5.30*10^−06^	5.37*10^−11^	6.9699
	RNN	12	5.52*10^−06^	5.67*10^−11^	7.1977	4.90*10^−06^	3.92*10^−11^	6.4344
	LSTM	3	6.05*10^−06^	6.16*10^−11^	7.9204	4.97*10^−06^	3.86*10^−11^	6.8457
	LSTM	12	5.37*10^−06^	4.99*10^−11^	7.1972	3.84*10^−06^	3.08*10^−11^	4.9881
HC	BPNN	2	1.03*10^−06^	1.83*10^−12^	10.5562	1.42*10^−06^	3.11*10^−12^	9.5433
	BPNN	12	8.21*10^−07^	1.26*10^−12^	7.9427	9.74*10^−07^	1.98*10^−12^	6.9851
	RNN	2	1.26*10^−06^	2.57*10^−12^	13.5108	1.24*10^−06^	2.93*10^−12^	8.9051
	RNN	12	1.12*10^−06^	2.11*10^−12^	11.3992	9.23*10^−07^	1.25*10^−12^	6.0301
	LSTM	2	1.37*10^−06^	3.29*10^−12^	13.46	6.76*10^−06^	4.85*10^−11^	44.6704
	LSTM	12	9.00*10^−07^	1.54*10^−12^	9.0873	8.84*10^−07^	1.98*10^−12^	5.8519
HE	BPNN	1	2.75*10^−07^	1.39*10^−13^	18.8614	2.22*10^−07^	9.83*10^−14^	11.571
	BPNN	12	2.17*10^−07^	9.94*10^−14^	13.1658	2.06*10^−07^	6.35*10^−14^	11.2271
	RNN	1	2.62*10^−07^	1.22*10^−13^	17.2014	2.61*10^−07^	1.66*10^−13^	13.3623
	RNN	12	2.54*10^−07^	1.34*10^−13^	15.8489	1.83*10^−07^	5.57*10^−14^	10.1930
	LSTM	1	2.45*10^−07^	1.06*10^−13^	16.2124	2.18*10^−07^	8.79*10^−14^	11.4885
	LSTM	12	2.73*10^−07^	1.46*10^−13^	16.7860	1.40*10^−07^	4.06*10^−14^	7.3504
HU	BPNN	12	2.23*10^−07^	1.19*10^−13^	7.9227	2.52*10^−07^	7.68*10^−14^	25.9586
	RNN	12	3.41*10^−07^	2.49*10^−13^	11.9683	1.35*10^−07^	2.36*10^−14^	13.3907
	LSTM	12	3.01*10^−07^	1.97*10^−13^	10.4429	1.61*10^−07^	3.53*10^−14^	16.6552

Note: *lookback* is the number of neurons in the input layer of BPNN, RNN and LSTM neural networks, which is a parameter that presents the number of values in each row as in Eq (12).

## Discussion

The internet-based infectious disease surveillance system of China has been used for over 10 years since it was created. As the data listed in Table 1, 20 924 951 cases and 11 892 deaths were reported in the system from 2005 to 2018. The disease data shows the change of each Hepatitis. For HA during 2005 to 2018, the case is from 76 102 to 16 736 that the case number was decreased about 78%, the deaths is from 36 to 4. Accompany the economic and medical development in China, the cases and deaths of HA show a significant downward trend since HA virus is usually spread by infected feces. During 2005 to 2018, the case of HB is around 1 200 000 for each year, since HB is commonly transmitted via infective blood, semen, pregnancy and childbirth, etc. The population and the number of new born baby have been a big number. It’s worth noting that from 2017, the cases and deaths increase a lot, which might be influenced by Two-child policy. For HC, the cases are increased more than 4 times in 2018 compared with 2005, since HC is spread primarily by blood-to-blood contact (injection drug, needlestick injuries, etc.) and the expansion of medical services. The deaths are a relatively stable quantity, which might be influenced by medical quality. For HE, the cases increase 47.69 percent from 2005 to 2018, while the death rate reduced from 0.29 percent to 0.05 percent, which might be influenced by the improvement of medical quality.

For infectious disease control and prevention, early awareness of the behaviors is significant, while the performance of statistical models in predicting future infectious disease incidence has been turned out helpful. There were some artificial neural network models applied to predict Hepatitis disease. There have been many time series, neural network models, to predict infectious disease incidence and death trends. How to choose the best model for the prediction of infectious disease has been attracting more and more attention. There is much research on comparing the different neural network model accuracy to predict infectious disease behavior, while different models have an inconsistent performance for prediction. For predicting Hepatitis A disease incidence trends, the conventional multiple-layer neural network model performs better than radial basis neural networks and time-delayed neural networks [22]. Many researchers are recommending that it is requisite to compare different forecasting models to predict the infectious behavior for different infectious diseases. In this paper, a rigorous study of three-time series neural network models was carried out with comparison to predict the pattern of Hepatitis incidence and death involving BPNN, RNN, LSTM. A comprehensive comparison among the three neural network models is illustrated from both principle and practical application.

In theory, the time series models of artificial neural networks capture the data information via nonlinear functions, which can approximate any continuous measurable function. In practice, BPNN is a feed-forward artificial neural network, which is based on the algorithm of backpropagation. RNN is a type of artificial neural network for recognizing patterns in time-series data, while the output depends on the sequence of time-series data other than a single piece of data. LSTM is a modified RNN architecture to address the vanishing and exploding issues of gradients and solve the problem of training over long sequences and retaining memory. Neural networks are nonparametric nonlinear models utilizing fewer prior assumptions based on the data generated by the intrinsic process. Thus, these neural network methods are more tolerant and less susceptible to predict time-series models compared with the conventional methods. There are many artificial neural network models widely used as powerful methods of modeling complex nonlinear and dynamic systems in various kinds of research areas.

In this paper, three different types of neural network models are employed to predict Hepatitis incidence, but they have different accuracy and efficacies compared via MAE, MAPE and MSE empirical measures, while the performance of the three neural network models shows their abilities to predict Hepatitis incidence. The LSTM accurately captured all Hepatitis training data compared with BPNN and RNN. The LSTM has the best performance to predict the disease incidence of Hepatitis B, Hepatitis C and Hepatitis E, while BPNN is the best model among the three to forecast the disease incidence of Hepatitis (involving the whole Hepatitis diseases), Hepatitis A and Hepatitis U.

In conclusion, we presented three artificial neural networks time series models on Hepatitis, which have the potential ability to predict the trends of time-series data due to the strong nonlinear mapping ability, especially when there exists a nonlinear relationship among the time series data. These methods can be potentially applied in time series data of other public health and clinical research, which would significantly promote Hepatitis disease control and management.

There are still some limitations to this research. The Hepatitis data obtained is started from 2005 since the government system is established in 2004. Therefore, the short time-series data of Hepatitis might influence the accuracy of the three neural network models. Additionally, it is difficult to explain clearly how the specific nonlinear functions work in the neural networks due to their black-box property. Furthermore, in this study, the comparative prediction accuracies are established only for Hepatitis diseases, while their findings might not suitable for other diseases.

Hepatitis diseases have a significant influence on people’s health. Advanced strategy with accurate estimation from the government can be made out quickly and efficiently. For further research, more comprehensive predicting theories and techniques should be researched in practice.

## Conclusion

This investigation used 14 years of nationally representative Hepatitis data to construct deep learning models to predict the incidence of Hepatitis based on the monthly incidence of Hepatitis through a national public health surveillance system in China mainland. We presented three deep learning methods, which show their significance to forecast the Hepatitis incidence and have the potential to assist the decision-makers in making efficient decisions for the early detection of the disease incidents, which would significantly promote Hepatitis disease control and management.
